# Effects of cow’s milk beta-casein variants on symptoms of milk intolerance in Chinese adults: a multicentre, randomised controlled study

**DOI:** 10.1186/s12937-017-0275-0

**Published:** 2017-10-25

**Authors:** Mei He, Jianqin Sun, Zhuo Qin Jiang, Yue Xin Yang

**Affiliations:** 1 Beijing Research Institute for Nutritional Resources, Beijing, China; 2 Clinical Nutrition Center, Huadong Hospital Affiliated to Fudan University, Shanghai, China; 3 Department of Nutrition, School of Public Health, Sun Yat-Sen University, Guangzhou, China; 4 Department of Food Nutrition, National Institute for Nutrition and Health, Chinese Center for Disease Control and Prevention, Beijing, China; 5 Chinese Nutrition Society, 6# Guang An Men Nei. Street, Fenghua Square, Building A, Unit 5, Room 1601/1602, Xuanwu District, Beijing, 100053 People’s Republic of China

**Keywords:** Beta-casein, Lactase, Lactose, Intolerance

## Abstract

**Background:**

A major protein component of cow’s milk is β-casein. The most frequent variants in dairy herds are A1 and A2. Recent studies showed that milk containing A1 β-casein promoted intestinal inflammation and exacerbated gastrointestinal symptoms. However, the acute gastrointestinal effects of A1 β-casein have not been investigated. This study compared the gastrointestinal effects of milk containing A1 and A2 β-casein versus A2 β-casein alone in Chinese adults with self-reported lactose intolerance.

**Methods:**

In this randomised, crossover, double-blind trial, with a 3-day dairy washout period at baseline, subjects were randomised to consume 300 mL of milk containing A1 and A2 β-casein (ratio 58:42; conventional milk) or A2 β-casein alone; subjects consumed the alternative product after a 7-day washout period. Urine galactose was measured at baseline after a 15 g lactose load. Subjects completed 9-point visual analogue scales for gastrointestinal symptoms (borborygmus, flatulence, bloating, abdominal pain, stool frequency, and stool consistency) at baseline and at 1, 3, and 12 h after milk consumption.

**Results:**

A total of 600 subjects were included. All six symptom scores at 1 and 3 h were significantly lower after consuming A2 β-casein versus conventional milk (all *P*<0.0001). At 12 h, significant differences remained for bloating, abdominal pain, stool frequency, and stool consistency (all *P*<0.0001). Symptom scores were consistently lower with A2 β-casein in both lactose absorbers (urinary galactose ≥0.27 mmol/L) and lactose malabsorbers (urinary galactose <0.27 mmol/L).

**Conclusion:**

Milk containing A2 β-casein attenuated acute gastrointestinal symptoms of milk intolerance, while conventional milk containing A1 β-casein reduced lactase activity and increased gastrointestinal symptoms compared with milk containing A2 β-casein. Thus, milk-related gastrointestinal symptoms may result from the ingestion of A1 β-casein rather than lactose in some individuals.

**Trial registration:**

NCT02878876, registered August 16, 2016. Retrospectively registered.

**Electronic supplementary material:**

The online version of this article (10.1186/s12937-017-0275-0) contains supplementary material, which is available to authorized users.

## Background

β-casein is a major protein component of cow’s milk, and numerous variants have been described, including the A1 and A2 types. The A1 and A2 types differ in terms of the amino acid at position 67, being histidine in the A1 type and proline in A2 β-casein. The presence of histidine in the A1 type increases the protein’s susceptibility to cleavage of the preceding seven amino acids, yielding β-casomorphin-7 (BCM-7), an exorphin with moderate agonistic activity on μ-receptors [1–3]. The knowledge that A1 β-casein may yield a functionally active exorphin has propelled research to understand whether A1 β-casein and BCM-7 affect gastrointestinal function or cause/exacerbate gastrointestinal inflammation in cell/animal models [4–9] and in people with milk or lactose intolerance [10, 11].

Thus far, however, very few studies have compared the effects of A1 and A2 β-casein on the gastrointestinal system in humans. In one recent study by Jianqin *et al*., 45 Chinese adults with self-reported intolerance to milk were randomised to receive milk containing either A2 β-casein or milk containing A1 and A2 β-casein (ratio 40:60; conventional milk) in a double-blind, 2×2 crossover manner with a washout period of 2 weeks before the study and between each phase [11, 12]. In that study, consumption of conventional milk was associated with increases in symptoms of post-dairy digestive discomfort and longer gastrointestinal transit times compared with milk containing only A2 β-casein. Consumption of conventional milk was also associated with increased small intestine inflammation and increased serum inflammatory biomarkers, as well as increased serum BCM-7 concentrations. In addition, when the subjects were divided according to their lactase activity based on urinary galactose tests, subjects with lactase deficiency reported greater gastrointestinal disturbances after consuming conventional milk compared with subjects with normal lactase activity, and that neither subgroup reported worsening of symptoms when they consumed milk containing A2 β-casein. Lactase deficiency was confirmed based on the subject’s reduced ability to metabolise lactose.

The results of this study prompted the hypothesis that the acute symptoms of milk intolerance (including self-reported lactose intolerance) in some people might be related to the presence of A1 β-casein in cow’s milk and that eliminating A1 β-casein could avoid these symptoms. Accordingly, the present study was designed to further evaluate the results of the study by Jianqin *et al*. [11, 12] by enrolling a much larger sample of subjects with self-symptoms of milk intolerance. Our primary objective was to compare the acute effects of milk containing either A2 β-casein or both A1 and A2 β-casein (conventional milk) on gastrointestinal symptoms in subjects with self-reported lactose intolerance and gastrointestinal discomfort. Our secondary objective was to examine whether these symptoms were correlated with lactase activity or the ability of these subjects to metabolise lactose.

## Methods

### Ethics

This three-centre, two-way parallel-group, crossover, randomised, double-blind controlled study was conducted in accordance with the Declaration of Helsinki (1996), Good Clinical Practice, and applicable regulatory requirements. The protocol was approved by the central institutional review board of the Shanghai Nutrition Society. All subjects provided written informed consent before study entry. The trial was registered on ClinicalTrials.gov with the ID NCT02878876.

### Subjects

Subjects were enrolled by the Chinese National Nutrition Society across three sites (Beijing, Guangzhou, and Shanghai). Males or females aged 20–50 years with self-reported lactose intolerance and digestive discomfort after consuming traditional milk were eligible for this study. Subjects were excluded if they had any of the following: any eating disorder, metabolic and/or chronic disease that was deemed likely to interfere with the study outcome measures, other than lactose intolerance; acute infection or gastroenteritis at time of enrolment; gastrointestinal disease likely to interfere with the study outcome measures, such as gastroesophageal reflux disease, irritable bowel disease, or Crohn’s disease; known allergy to cow’s milk products; doctor-diagnosed immunodeficiency and any severe disease. Because the incidence of lactose intolerance may vary with age, we planned to enrol approximately similar numbers of subjects in two age groups: 20–35 and 36–50 years.

### Study products

Milk containing either A1 and A2 β-casein (conventional milk) or only A2 β-casein was provided by The a2 Milk Company (Shanghai, China). Both products were confirmed to be identical (Table 1), except for the β-casein content. The ratio of A1 β-casein to A2 β-casein in conventional milk was 58:42. A1 and A2 β-casein levels in the study milk were measured by an independent laboratory (Analytica Laboratories, Ruakura, New Zealand) using ultra-high-performance liquid chromatography–diode array detector–tandem mass spectrometry.

**Table 1:** Nutritional composition of the two types of milk

Nutrient (per 100 mL)	A2 milk	A1 milk
Energy (kJ)	278	270
Protein (g)	3.3	3.3
Fat (g)	3.7	3.5
Saturated fat (g)	2.4	2.1
Carbohydrate (g)	5	4.8
Sodium (mg)	37	45
Calcium (mg)	117	120
Lactose	5	4.8

*A1* conventional milk containing A1 and A2 β-casein, *A2* milk containing A2 β-casein

### Study design

**Fig. 1:**
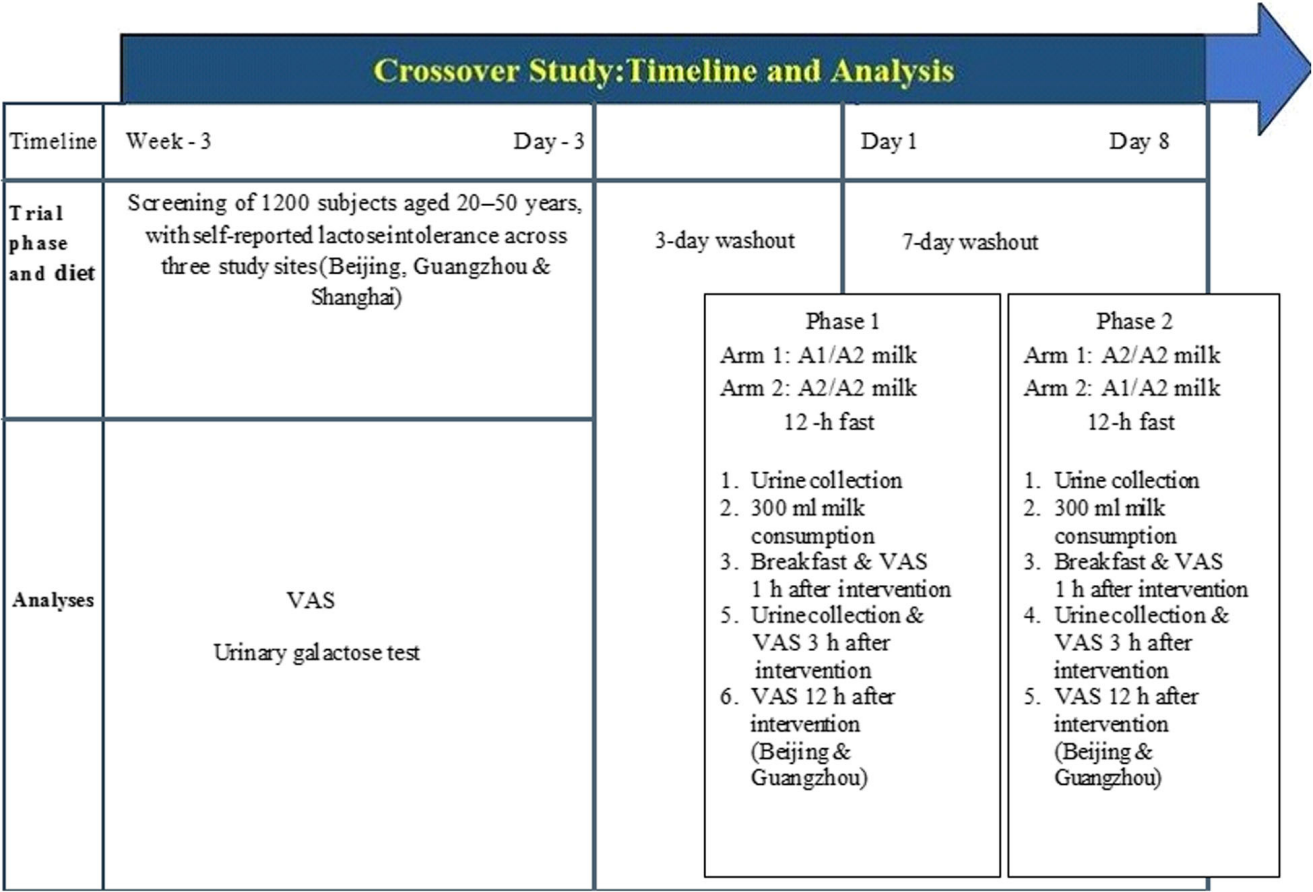
Study design. VAS, visual analogue scale

Figure 1 shows the design of the study. After a screening period, eligible subjects underwent urinary galactose tests and completed a visual analogue scale (VAS) to assess their baseline gastrointestinal symptoms [13]. Subjects were then randomized to one of two treatment sequences in which they received conventional milk on Day 1 and milk containing A2 β-casein on Day 8, or vice versa.

Randomisation was performed using a computer-generated list prepared on site. Subjects were stratified by age group (20–35 years/36–50 years) to either sequence 1 (A1/A2➔A2) or sequence 2 (A2➔A1/A2) according to the allocation numbers prepared in sealed envelopes. The allocation list was generated using SAS statistical software (SAS Institute, Cary, NC, USA).

Subjects returned to the study site on Day 1 after a 3-day washout of dairy products and a 12-h overnight fast. The subjects were instructed to avoid dairy products during the washout, but were not provided any non-dairy milk products during this time. At the study site, the subjects provided a urine sample and consumed 300 mL of the allocated milk type in accordance with the allocation schedule.

All milk products were provided in a double-blind manner, and subjects used a food diary to record milk intake and adherence to each intervention. In Beijing, the study products were prepared and repackaged by technicians assigned by the principal investigator. At the other sites, the study products were prepared by the sponsor. All products were identical in packaging and were only labelled with the subject’s identification number.

At 1 h after consuming the milk product, the subjects ate breakfast and completed the VAS for gastrointestinal symptoms. The breakfast was congee and a steamed bun in Beijing, and comprised fried chicken, congee and bread in Shanghai and Guangzhou. At 3 h, subjects provided a urine sample and completed the VAS for gastrointestinal symptoms. After completing these assessments, the subjects could leave the study site. At 12 h, subjects in Beijing and Guangzhou completed the VAS for gastrointestinal symptoms. This VAS was completed via a telephone call. The 12-h follow up assessment was not possible at Shanghai owing to limited resources and logistical constraints.

The subjects continued a dairy-free diet for 7 days and, on Day 8, they returned to the study site to repeat the study procedures, and this time they consumed the opposite milk product. The subjects were also asked to complete food frequency questionnaires to assess dietary adherence.

### Objectives

The primary objective was to compare the effects of consuming milk containing solely the A2 type of β-casein with conventional milk containing both A1 and A2 types of β-casein on acute self-recorded lactose intolerance and gastrointestinal discomfort occurring within several hours of consuming milk. Therefore, the primary endpoint was gastrointestinal symptom scores assessed by VAS. Secondary objectives included the following: (1) to compare the effects of both milk products on lactase activity, which was assessed in terms of urinary galactose after an oral lactose load; (2) to compare/contrast the shifts in lactase activity induced by the type of β-casein with self-reported symptoms of lactose intolerance; and (3) to determine whether age was correlated with the symptoms of milk intolerance or urinary galactose, the subjects were also divided into two age groups (20–35 years and 36–50 years). Consequently, urinary galactose, as a surrogate marker of lactase activity, was included as a secondary endpoint.

### Gastrointestinal symptom scores

Baseline gastrointestinal symptoms were evaluated before product intervention by asking the subjects to report their symptoms at the last time they consumed milk. The following gastrointestinal symptoms were assessed by the VAS: borborygmus, flatulence, bloating, abdominal pain, stool frequency, and stool consistency. Each symptom was assessed using a 9-point scale, where 0 = not at all and 9 = very serious. Improvements in gastrointestinal symptoms after consumption of milk containing A2 β-casein relative to conventional milk were then classified as follows: no symptoms; significant improvement in symptoms (reduction in score of ≥4 for that symptom); slight improvement in symptoms (reduction in score of 1 to ≤3 for that symptom); or no difference or worsening in symptoms (no change in score or an increase in the score).

### Food frequency questionnaire

Subjects were asked to complete food frequency questionnaires (Additional file 1: Table S1) to document adherence to the dairy-free diet during the 3-day and 7-day washout periods. The questionnaires were conducted by the investigators on days 1 and 8 at each study site. The questionnaires recorded the food intake in the previous 24 h. The subjects also confirmed that no dairy product was consumed during the washout period.

### Urinary galactose

Urinary galactose was measured as an indicator of lactase deficiency using a fluorescence spectrum analysis method. To assess the effects of lactose malabsorption on gastrointestinal symptoms, the subjects were divided into two subgroups according to their change in urinary galactose at 3 h after ingestion of 15 g of lactose at baseline (300 mL of conventional milk). Individuals with a urinary galactose concentration <0.9 mmol/L after a 50-g oral lactose intake are considered lactose malabsorbers [14, 15]. Therefore, we used 0.9*(15/50)=0.27 mmol/L as the threshold to distinguish lactose absorbers from malabsorbers.

### Adverse events

Adverse events occurring any time between enrolment and up to 30 days last assessment were to be recorded. The subjects were asked to report adverse events via telephone or at each visit. The adverse events were classified in terms of their seriousness and relationship with the study product by the subject’s investigator.

### Statistics

#### Sample size

Based on our clinical experience together with data published in previous studies, it was determined to enrol approximately 630 subjects (210 at each participating site) to achieve a total of 600 subjects after accounting for a dropout rate of 5%. The sample size was estimated based on prior studies [11] reporting a confidence of *p* < 0.05 for upper gastrointestinal inflammation and the greater sample size of our study was determined to be sufficient to examine gastrointestinal symptoms putatively linked to milk consumption. Enrolment was stopped once the planned sample size had been enrolled.

#### Data analysis

Baseline characteristics and adverse events are summarised descriptively in terms of the mean and standard deviation, and number (%) of subjects. Gastrointestinal symptoms were analysed using generalised estimating equations (GEE) for ordinal repeated measures, with fixed effects of study product (A1 or A2), study visit (1 or 2), and age group (20–35 or 36–50 years old), a random subject effect nested within the sequence of study treatment (A1➔A2 or A2➔A1), and adjusted for baseline symptom scores. Contrast tests were generated to compare means for each product. Urinary galactose concentrations were analysed using mixed effects analysis of variance with fixed effects of study product (A1 or A2), study visit (1 or 2) and age group (20–35 or 36–50 years old), a random subject effect nested within the sequence of study treatment (A1➔A2 or A2➔A1), and adjusted for baseline symptom scores. Type III tests of fixed effects were used to test the effect of study products. Contrast tests were generated to compare means for each product. Differences in gastrointestinal symptoms and urinary galactose concentrations were analysed using the Kruskal–Wallis test and analysis of variance, respectively.

Considering the geographical (environmental and lifestyle) differences between the three sites, and because the VAS for gastrointestinal symptoms was not assessed at 12 h in Shanghai, it was planned to analyse the data for all three sites individually and then by all three sites combined.

The residual plots for all VAS variables showed no violations of normality for data at 1 and 3 h, although some skewness was apparent at 12 h. Accordingly, all VAS scores are presented as the median (range).

## Results

### Subjects

The first subject was enrolled on January 18, 2016 (Shanghai), and the last subject completed the study on April 19, 2016 (Beijing). A total of 1200 subjects were initially screened and 642 started the study, 230 in Beijing, 210 in Guangzhou, and 202 in Shanghai. In Beijing, 13 subjects discontinued the study and questionnaires were not completed by 17 subjects. In addition, 10 subjects in Guangzhou and 2 in Shanghai discontinued the study. Therefore, data were available for 600 subjects (200 per site). The characteristics of participating subjects are summarized in Table 2. All of the subjects were non-regular milk drinkers and had not consumed milk for at least 1 month before enrolment. However, some subjects reported that they consumed yoghurt up to twice a week (120 mL per time). The results from the food frequency questionnaire revealed that all subjects that completed the study showed adherence to a dairy-free diet during the washout periods.

**Table 2:** Subject characteristics

Site	Beijing				Guangzhou				Shanghai			
Sequence	A1➔A2 (*n*=100)		A2➔A1 (*n*=100)		A1➔A2 (*n*=100)		A2➔A1 (*n*=100)		A1➔A2 (*n*=100)		A2➔A1 (*n*=100)	
	n/mean	%/SD	n/mean	%/SD	n/mean	%/SD	n/mean	%/SD	n/mean	%/SD	n/mean	%/SD
Males	56	56%	48	48%	39	39%	41	41%	52	52%	43	43%
Age (years)	37.2	8.3	35.5	8.5	36	7.6	36.3	7.6	35	8.9	35	9.3
20–35 years	50	50%	50	50%	50	50%	50	50%	50	50%	50	50%
	30	4.6	28.3	4.2	39.8	4.4	30.1	4.3	27.3	5	26.9	4.1
36–50 years	50	50%	50	50%	50	50%	50	50%	50	50%	50	50%
	44.3	3.7	42.7	4.8	42.2	4.2	42.5	4.4	42.6	3.8	43.2	4.6
Weight (kg)	69.7	11.5	68.7	11.3	60.1	10.4	60.4	11.2	62.7	12.1	63.5	11.3
Height (cm)	171.1	10	168.7	9.6	164.5	7.0	163.6	7.4	166.9	7.9	164.2	12
Site	All sites											
Sequence	A1➔A2 (*n*=300)		A2➔A1 (*n*=300)									
	n/mean	%/SD	n/mean	%/SD								
Males	147	49%	132	44%								
Age (years)	36	8.3	35.6	8.5								
20–35 years	150	50%	150	50%								
	29	4.8	28.4	4.4								
36–50 years	150	50%	150	50%								
	43	4	42.8	4.6								
Weight (kg)	64.2	12	64.2	11.7								
Height (cm)	167.5	8.8	165.5	10.1								

*A1* conventional milk containing A1 and A2 β-casein, *A2* milk containing A2 β-casein

### Gastrointestinal symptoms

There were no significant effects of visit or age group on symptom scores (Additional file 1: Table S2). Therefore, data are grouped according to the intervention received. The gastrointestinal symptom VAS scores recorded are summarised according to the study intervention in Table 3. As indicated in this table, the symptom scores for all six symptoms were consistently lower with milk containing A2 β-casein than with conventional milk at both 1 and 3 h after consumption (all *P<*0.0001). The differences in symptom scores between the two products were also observed at all three sites separately (Additional file 1: Table S3). Differences in symptom scores were still apparent at 12 h for bloating, abdominal pain, stool frequency, and stool consistency in Beijing and for abdominal pain, stool frequency, and stool consistency in Guangzhou.

**Table 3:** Gastrointestinal symptom scores (all study sites combined)

Variable	Time	Visit 1 (baseline)				Visit 2				GEE analysis		
		A1 (*n*=300)^a^		A2 (*n*=300)^a^		A1 (*n*=300)^a^		A2 (*n*=300)^a^		Product (A2 vs. A1)		
		Median	Range	Median	Range	Median	Range	Median	Range	Estimate	SE	P
Borborygmus	1 h	3	0–6	2	0–6	3	0–6	2	0–6	1.327	0.097	<0.0001
	3 h	3	0–6	2	0–6	3	0–6	2	0–5	1.912	0.103	<0.0001
	12 h	0	0–2	0	0–2	0	0–2	0	0–2	0.134	0.180	0.458
Flatulence	1 h	2	0–6	2	0–5	3	0–6	1	0–5	1.516	0.098	<0.0001
	3 h	3	0–6	2	0–5	3	0–6	2	0–4	1.869	0.101	<0.0001
	12 h	0	0–3	0	0–3	0	0–3	0	0–2	0.245	0.130	0.059
Bloating	1 h	2	0–6	1	0–6	3	0–6	1	0–5	1.474	0.098	<0.0001
	3 h	3	0–6	2	0–5	3	1–6	2	0–4	1.922	0.096	<0.0001
	12 h	2	0–5	1	0–4	2	0–4	1	0–3	0.883	0.126	<0.0001
Abdominal Pain	1 h	0	0–5	0	0–5	1	0–5	0	0–3	0.714	0.082	<0.0001
	3 h	2	0–5	1	0–4	2	0–6	0	0–3	1.903	0.101	<0.0001
	12 h	2	0–4	1	0–3	2	0–5	1	0–3	1.887	0.124	<0.0001
Stool Frequency	1 h	0	0–5	0	0–4	0	0–5	0	0–3	0.749	0.088	<0.0001
	3 h	2	0–5	0	0–3	2	0–5	0	0–3	2.276	0.110	<0.0001
	12 h	1	0–2	0	0–2	1	0–3	0	0–2	1.778	0.156	<0.0001
Stool Consistency	1 h	0	0–5	0	0–4	0	0–5	0	0–2	0.799	0.092	<0.0001
	3 h	2	0–5	0	0–4	2	0–5	0	0–2	2.375	0.115	<0.0001
	12 h	1	0–3	0	0–2	1	0–3	0	0–2	2.051	0.149	<0.0001

*A1* conventional milk containing A1 and A2 β-casein, *A2* milk containing A2 β-casein, *GEE* generalized estimating equation, *SE* standard error

^a^n=400 at 12 h (the VAS was not assessed at 12 h in Shanghai)

Significant between-group differences in gastrointestinal symptoms were also observed at 1 and 3 h between the two groups when data for all three sites were combined (all *P*<0.0001) (Table 3). At 12 h, significant differences remained for bloating, abdominal pain, stool frequency, and stool consistency (all *P*<0.0001), but not flatulence (*P=*0.059) or borborygmus (*P*=0.458). These findings indicate that milk containing A2 β-casein was associated with less severe gastrointestinal symptoms within 1–12 h after consumption compared with conventional milk.

We also classified gastrointestinal symptom scores according to the direction of change at 1, 3, and 12 h relative to the baseline scores in terms of no symptoms, significant improvement, slight improvement, and no difference. As indicated in Additional file 1: Table S4 (subjects at each study site separately) and Table 4 (all subjects combined), there was a greater trend towards slight improvements in the gastrointestinal symptoms when the subjects consumed milk containing A2 β-casein as compared with their consumption of conventional milk.

**Table 4:** Proportions of subjects with improvements in gastrointestinal symptoms after consuming milk containing A2 β-casein relative to milk containing A1 β-casein (all study sites combined)

Measurement	Improvement	Borborygmus		Flatulence		Bloating		Abdominal Pain	
		n	%	n	%	n	%	n	%
1 h (*n*=600)	No symptom	50	8.3%	61	10.2%	112	18.7%	119	19.8%
	Significant improvement	9	1.5%	9	1.5%	6	1.0%	1	0.2%
	Slight improvement	310	51.7%	314	52.3%	279	46.5%	88	14.7%
	No difference	231	38.5%	216	36.0%	203	33.8%	392	65.3%
3 h (*n*=600)	No symptom	33	5.5%	29	4.8%	62	10.3%	240	40.0%
	Significant improvement	8	1.3%	9	1.5%	5	0.8%	6	1.0%
	Slight improvement	362	60.3%	341	56.8%	338	56.3%	131	21.8%
	No difference	197	32.8%	221	36.8%	195	32.5%	223	37.2%
12 h (*n*=400)^a^	No symptom	51	12.8%	102	25.5%	83	20.8%	121	30.3%
	Significant improvement	0	0.0%	0	0.0%	1	0.3%	0	0.0%
	Slight improvement	4	1.0%	24	6.0%	105	26.3%	140	35.0%
	No difference	345	86.3%	274	68.5%	211	52.8%	139	34.8%
Measurement	Improvement	Stool Frequency		Stool Consistency		All Symptoms			
		n	%	n	%	n	%		
1 h (*n*=600)	No symptom	120	20.0%	128	21.3%	16	2.7%		
	Significant improvement	1	0.2%	1	0.2%	282	47.0%		
	Slight improvement	65	10.8%	61	10.2%	236	39.3%		
	No difference	414	69.0%	410	68.3%	66	11.0%		
3 h (*n*=600)	No symptom	304	50.7%	310	51.7%	4	0.7%		
	Significant improvement	0	0.0%	1	0.2%	480	80.0%		
	Slight improvement	114	19.0%	87	14.5%	100	16.7%		
	No difference	182	30.3%	202	33.7%	16	2.7%		
12 h (*n*=400)^a^	No symptom	199	49.8%	202	50.5%	21	5.3%		
	Significant improvement	0	0.0%	0	0.0%	127	31.8%		
	Slight improvement	25	6.3%	22	5.5%	186	46.5%		
	No difference	176	44.0%	176	44.0%	66	16.5%		

^a^The VAS was not assessed at 12 h in Shanghai

### Effects of age on gastrointestinal symptoms

The effect of age on gastrointestinal symptoms was examined. As indicated in Additional file 1: Tables S2 and S5, age group did not have a significant impact on gastrointestinal symptoms when evaluated using GEE analysis or Kruskal–Wallis test.

### Urinary galactose concentrations

Table 5 shows the urinary galactose concentrations at baseline and at 3 h after the consumption of milk containing A2 β-casein or conventional milk. As would be expected after the consumption of milk, the urinary galactose concentrations increased between baseline and 3 h in both groups. However, the magnitude of the increase was significantly greater when the subjects consumed milk containing A2 β-casein than when they consumed conventional milk, with consistent changes in each site and in all subjects combined. Age group was not associated with differences in urinary galactose concentrations based on the GEE analysis (Additional file 1: Table S6)

**Table 5:** Urinary galactose concentrations after consumption of the study products

Site	Time	Visit 1 (baseline)				Visit 2				LSM difference		
		A1 (*n*=300)^a^		A2 (*n*=300)^a^		A1 (*n*=300)^a^		A2 (*n*=300)^a^		Product (A2 vs. A1)		
		Mean (mmol/L)	SD	Mean (mmol/L)	SD	Mean (mmol/L)	SD	Mean (mmol/L)	SD	Estimate	SE	P
Beijing	Baseline	0.72	0.30	0.71	0.30	0.70	0.31	0.71	0.28			
	3 h	0.84	0.33	1.06	0.39	0.81	0.31	1.07	0.42	0.243	0.034	<0.0001
	Change from baseline	0.12	0.27	0.35	0.43	0.11	0.31	0.36	0.47	0.244	0.038	<0.0001
Guangzhou	Baseline	0.72	0.32	0.73	0.33	0.73	0.38	0.70	0.38			
	3 h	0.85	0.49	1.01	0.39	0.96	0.58	1.07	0.69	0.142	0.044	0.002
	Change from baseline	0.13	0.40	0.28	0.32	0.23	0.46	0.37	0.59	0.144	0.045	0.002
Shanghai	Baseline	0.71	0.26	0.71	0.25	0.72	0.28	0.72	0.21			
	3 h	0.85	0.36	0.99	0.39	0.83	0.37	1.02	0.42	0.165	0.034	<0.0001
	Change from baseline	0.14	0.37	0.28	0.41	0.12	0.31	0.30	0.38	0.165	0.036	<0.0001
All sites	Baseline	0.72	0.29	0.72	0.30	0.72	0.32	0.71	0.30			
	3 h	0.85	0.40	1.02	0.39	0.87	0.44	1.05	0.53	0.183	0.022	<0.0001
	Change from baseline	0.13	0.35	0.30	0.39	0.15	0.37	0.34	0.49	0.184	0.023	<0.0001

*A1* conventional milk containing A1 and A2 β-casein, *A2* milk containing A2 β-casein, *LSM* least-squares mean, *SD* standard deviation, *SE* standard error

^a^n=400 at 12 h (the VAS was not assessed at 12 h in Shanghai)

### Relationship between malabsorption and gastrointestinal symptoms

Overall, 170 (28.3%) and 430 (71.7%) of subjects at all three sites combined were classified as lactose absorbers and lactose malabsorbers, respectively. The proportions of lactose absorbers and lactose malabsorbers were similar at all three sites separately (Beijing: 48 [24.0%] vs 152 [76.0%]; Guangzhou: 69 [34.5%] vs 131 [65.5%]; Shanghai: 53 [26.5%] vs 147 [73.5%]). For subjects in all sites combined, the gastrointestinal symptom scores were significantly lower for milk containing A2 β-casein as compared with conventional milk at both 1 and 3 h in lactose absorbers and lactose malabsorbers (all *P≤*0.0002) (Table 6). The symptom scores for bloating, abdominal pain, stool frequency, and stool frequency at 12 h after consumption were also significantly lower for milk containing A2 β-casein compared with conventional milk in both lactose absorbers and lactose malabsorbers (all *P*<0.0001). In addition, there were trends towards slight improvements in gastrointestinal symptoms after consuming milk containing A2 β-casein compared with conventional milk (Additional file 1: Table S7). Similar results were observed when the data were analysed for each site separately (data not shown).

**Table 6:** Effects of lactose malabsorption on gastrointestinal symptoms

1 h	Absorbers (*n*=170)							Malabsorbers (*n*=430)						
	A1		A2		GEE estimate			A1		A2		GEE estimate		
	Median	Range	Median	Range	Estimate	SE	P	Median	Range	Median	Range	Estimate	SE	P
Borborygmus	3	0–6	2	0–6	1.562	0.218	<0.0001	3	0–6	2	0–5	1.244	0.116	<0.0001
Flatulence	3	0–6	2	0–5	1.826	0.212	<0.0001	2	0–6	1	0–5	1.395	0.119	<0.0001
Bloating	2	0–6	1	0–5	1.698	0.201	<0.0001	2	0–6	1	0–6	1.406	0.12	<0.0001
Abdominal Pain	1	0–5	0	0–4	1.552	0.206	<0.0001	0	0–5	0	0–5	0.396	0.107	0.0002
Stool Frequency	1	0–5	0	0–4	1.328	0.212	<0.0001	0	0–5	0	0–3	0.518	0.121	<0.0001
Stool Consistency	1	0–5	0	0–4	1.339	0.207	<0.0001	0	0–5	0	0–4	0.566	0.128	<0.0001
3 h	Absorbers (*n*=170)							Malabsorbers (*n*=430)						
	A1		A2		GEE estimate			A1		A2		GEE estimate		
	Median	Range	Median	Range	Estimate	SE	P	Median	Range	Median	Range	Estimate	SE	P
Borborygmus	3	0–5	2	0–5	1.701	0.206	<0.0001	3	0–6	2	0–6	2.022	0.124	<0.0001
Flatulence	3	0–5	2	0–5	1.424	0.199	<0.0001	3	0–6	2	0–5	2.079	0.133	<0.0001
Bloating	3	0–5	2	0–5	1.617	0.196	<0.0001	3	0–6	2	0–4	2.066	0.125	<0.0001
Abdominal Pain	2	0–6	0	0–4	1.375	0.204	<0.0001	2	0–6	0	0–4	2.149	0.134	<0.0001
Stool Frequency	1	0–5	0	0–3	1.866	0.222	<0.0001	2	0–5	0	0–3	2.454	0.136	<0.0001
Stool Consistency	1	0–5	0	0–4	1.904	0.216	<0.0001	2	0–5	0	0–3	2.586	0.146	<0.0001
12 h^a^	Absorbers (*n*=117)							Malabsorbers (*n*=283)						
	A1		A2		GEE estimate			A1		A2		GEE estimate		
	Median	Range	Median	Range	Estimate	SE	P	Median	Range	Median	Range	Estimate	SE	P
Borborygmus	0	0–2	0	0–2	–0.479	0.375	0.202	0	0–2	0	0–2	0.317	0.211	0.133
Flatulence	1	0–3	0	0–3	0.165	0.251	0.509	0	0–3	0	0–2	0.297	0.159	0.062
Bloating	2	0–4	1	0–4	1.074	0.242	<0.0001	2	0–5	1	0–4	0.812	0.15	<0.0001
Abdominal Pain	2	0–3	1	0–3	2.239	0.253	<0.0001	2	0–5	1	0–3	1.793	0.156	<0.0001
Stool Frequency	1	0–3	0	0–2	1.618	0.282	<0.0001	1	0–2	0	0–2	1.854	0.192	<0.0001
Stool Consistency	1	0–2	0	0–1	1.988	0.291	<0.0001	1	0–3	0	0–2	2.077	0.183	<0.0001

*A1* conventional milk containing A1 and A2 β-casein, *A2* milk containing A2 β-casein

^a^The VAS was not assessed at 12 h in Shanghai

### Adverse events

Overall, 16 adverse events were reported during the study. Adverse events reported by subjects in Beijing were flu (2, 1.0%), croup (2, 1.0%), upper respiratory infection (3, 1.5%), anal fissure (1, 0.5%), urinary tract infection (1, 0.5%), and atopic dermatitis (1, 0.5%). Four subjects in Guangzhou reported adverse events, which were cough without other related symptoms (2, 1.0%), pneumonia (1, 0.5%), and bronchitis (1, 0.5%). Pneumonia (1, 0.5%) and torticollis (1, 0.5%) were reported in Shanghai. None of the adverse events were considered related to the study products, and none of the adverse events were classified as serious.

## Discussion

Ho *et al*. [10] and Jianqin *et al*. [11] performed preliminary studies to compare the effects of conventional milk and milk containing only A2 β-casein on gastrointestinal symptoms in humans. Ho *et al*. [10] revealed that milk containing A1 β-casein was associated with significantly softer stool showing higher consistency scores, as determined using the Bristol Stool Scale, compared with milk containing A2 β-casein. In addition, consumption of A1 β-casein milk was associated with increased faecal calprotectin, a marker of intestinal inflammation [16]. Meanwhile, Jianqin *et al*. [11] revealed that consumption of conventional milk was associated with greater symptoms of post-dairy digestive discomfort in subjects with self-reported lactose intolerance. The worsening of gastrointestinal symptoms was apparent in lactose tolerant and lactose intolerant subjects. A subsequent analysis [17] of the study by Jianqin et al. revealed increased concentrations of inflammatory biomarkers and BCM-7 after consumption of milk containing both β-casein types compared with consumption of milk containing only A2 β-casein. However, the studies by Ho *et al*. [10] and Jianqin *et al*. [11] were relatively small, involving 40 and 45 subjects, and warranted confirmation in larger-scale studies. Nevertheless, the results highlighted a link between A1 β-casein, gastrointestinal inflammation, and symptoms of milk intolerance. Notably, subjects confirmed to be lactose malabsorbers tolerate milk containing only A2 β-casein, even though the lactose level was similar to that of conventional milk, suggesting that the type of β-casein may contribute to the symptoms of lactose intolerance in some people.

Accordingly, the objectives of the present were to compare the effects of consuming milk containing either A2 β-casein or conventional milk containing both A1 and A2 β-casein on acute self-recorded lactose intolerance and gastrointestinal discomfort occurring within several hours of consuming milk. In addition, we sought to examine the effects of both milk products on lactase activity to determine if changes in lactase activity are linked to the changes in self-reported symptoms of milk intolerance. We also examined whether age was correlated with a shift in lactase activity and the symptoms of milk intolerance.

This cross-over study of 600 Chinese subjects with self-reported milk intolerance revealed significant differences in gastrointestinal symptoms after the consumption of milk containing A2 β-casein or conventional milk. Of note, the gastrointestinal symptom scores were significantly lower at 1, 3 and 12 h after consumption of milk containing A2 β-casein relative to the consumption of conventional milk. These results suggest that elimination of A1 β-casein from the diet was associated with reduced severity of acute gastrointestinal symptoms after milk intake in this population.

It is important to note that the baseline symptoms were evaluated before consumption of either milk product by asking the subjects to report their symptoms at the last time they consumed milk. Accordingly, the subjects possibly recalled their worst experience. To avoid this potential source of bias, the analyses of gastrointestinal symptoms were adjusted for baseline scores to account for individual differences.

The exact mechanism by which acute exposure to A1 β-casein augments gastrointestinal symptoms relative to exposure to A2 β-casein is unclear, but we speculate that inflammation might be a contributing factor. This is supported by the studies by Ho *et al*. [10], Deth *et al*. [17], and Trivedi *et al*. [18] who noted increases in the concentrations of inflammatory biomarkers following exposure to A1 β-casein. However, these studies involved longer durations of exposure than our study, in which symptoms were assessed up to 12 h after exposure. To our knowledge, no studies have examined the acute effects of A1 β-casein exposure on gastrointestinal inflammation in humans.

Although no studies have examined the acute effects of A1 β-casein, some studies have investigated the acute effects of other dietary proteins on inflammatory biomarkers.

For example, Kristjánsson *et al*. [19] investigated mucosal inflammatory reactivity to cow’s milk protein and wheat gluten in 20 patients with coeliac disease and 15 healthy controls. The mucosal reactions to these proteins were assessed 15 h after exposure. Of note, the gluten challenge induced neutrophil activation and nitric oxide synthesis. Ten patients showed strong inflammatory reactions to cow’s milk protein. Six patients sensitive to cow’s milk were also challenged with casein and α-lactalbumin. In this experiment, casein induced an inflammatory response similar to that elicited by cow’s milk. These findings suggest that casein elicits an inflammatory response similar to that elicited by gluten in patients with coeliac disease. These results are consistent with the study by Trivedi *et al*. [18] who reported that A1 β-casein-derived BCM-7 and gluten-derived exorphin share a mechanistic pathway for inducing oxidative stress in cultured human gut epithelial cells and neuronal cells.

Holmer-Jensen *et al*. [20] conducted a randomized crossover study in which 11 obese non-diabetic subjects consumed a fat-rich mixed meal containing cod protein, whey isolate, gluten, or casein. They observed some differences in the acute effects of dietary protein on postprandial inflammatory biomarkers. Intriguingly, all four proteins were associated with reductions in monocyte chemoattractant protein-1 and increases in CCL5/RANTES. The whey protein meal was associated with the smallest reduction in monocyte chemoattractant protein-1 and the largest increase in CCL5/RANTES compared with the other meals.

Pal and Ellis [21] compared the effects (within 6 h) of whey protein, caseinate, and glucose on blood pressure, vascular function, and inflammatory markers in 20 overweight and obese postmenopausal women. Although systolic blood pressure, diastolic blood pressure, and augmentation index decreased initially after each meal, there were no significant differences in these variables between the glucose, casein, or whey groups. Moreover, they found no differences in plasma inflammatory markers.

Finally, Nestel *et al*. [22] found no changes in systemic inflammatory and atherogenic biomarkers after ingestion of a variety of dairy products (low-fat milk, or 45 g fat from butter, cream, yoghurt, or cheese) in 12 overweight subjects after a single meal. Moreover, in a 4-week study of 12 subjects who consumed 50 g dairy fat daily as either butter, cream and ice cream (non-fermented) or cheese plus yoghurt (fermented) dairy foods, there were no apparent differences in fasting biomarker concentrations between the non-fermented and fermented dairy products.

Unfortunately, none of these studies assessed gastrointestinal symptoms and changes in plasma inflammatory markers might not be correlated with local inflammation.

Nevertheless, the results of these studies suggest that dietary proteins might have differential effects on gastrointestinal inflammation, and further studies might be necessary to examine whether changes in localised gastrointestinal inflammation are correlated with gastrointestinal symptoms.

It is also important to consider that lactose might contribute to the gastrointestinal symptoms in this cohort of subjects with self-reported lactose intolerant. Indeed, an increase in gastrointestinal symptoms was observed when the subjects consumed conventional milk. However, the symptoms were reduced when the subjects consumed milk containing only A2 β-casein, indicating that A1 β-casein-induced inflammation may be linked to the symptoms of lactose intolerance.

To examine the impact of lactose malabsorption on gastrointestinal symptoms, we divided the subjects as lactose absorbers and lactose malabsorbers, based on the results of the urinary galactose test. Of note, the gastrointestinal symptoms after consumption of milk containing A2 β-casein were comparable between the lactose absorbers and lactose malabsorbers.

Based on these findings, we propose the hypothesis that the gastrointestinal symptoms in some subjects with self-reported lactose intolerance might be related to A1 β-casein rather than lactose itself. This seems feasible considering that the lactose concentrations were comparable in both milk products.

We also explored the possibility that age had an impact on gastrointestinal symptoms or the correlation between lactose malabsorption and gastrointestinal symptoms. As indicated in Additional file 1: Tables S2, S5 and S6, age was not significantly associated with gastrointestinal symptoms. However, because the upper age range was limited to 50 years, it is possible that older subjects might experience more severe gastrointestinal symptoms after dairy intake.

The results of this study should be interpreted with care, considering the limitations of this study, especially in terms of the mechanistic link between the observed compromise in lactose digestion and the type of β-casein. In addition, we used an indirect method to assess lactase activity. Last, the consumption of other non-dairy foods and drinks by subjects is a potential confounder; however, rather than deny intake of food to participants, we ensured that any foods and drinks consumed were dairy-free and that there was consistency in food intake type for both interventions. Further studies are warranted to examine the putative role of A1 β-casein in gastrointestinal inflammation, the effects of inflammation on the expression and/or activity of lactase enzyme, and the proportion of people with lactose intolerance who would benefit from excluding A1 β-casein from their diet. Additionally the effects of long-term exposure to milk in terms of the changes in gastrointestinal health need to be examined in future trials, and whether chronic exposure conditions desensitise the gastrointestinal tract to A1 β-casein consumption.

## Conclusions

In conclusion, this study showed that consumption of milk containing A2 β-casein attenuated the acute gastrointestinal symptoms following milk intake relative to conventional milk containing A1 and A2 β-casein in Chinese subjects with self-reported lactose intolerance. Gastrointestinal symptoms after consuming milk containing A2 β-casein were consistently reduced in both lactose absorbers and lactose malabsorbers. These findings suggest that, in some individuals with self-reported lactose intolerance, the adverse gastrointestinal symptoms following milk intake might be related to the presence of A1 β-casein in milk rather than lactose itself.

## Additional file

Additional file 1:
**Table S1.** Food frequency questionnaire. **Table S2.** Gastrointestinal symptom scores at each study site: GEE analysis of visit and age group. **Table** **3.** Gastrointestinal symptom scores in individual study sites**. Table** **4.** Proportions of subjects with improvements in gastrointestinal symptoms at each study site. **Table** **5.** Differences in gastrointestinal symptoms between the two age groups by study product (all study sites combine). **Table** **6.** Urinary galactose concentrations after consumption of the study products: LSM difference for visit and age group. **Table 7.** Proportions of subjects with improvements in gastrointestinal symptoms according to lactose absorption/malabsorption. (XLSX 37 kb)

## Abbreviations

BCM-7: Beta-casomorphin 7
GEE: Generalised estimating equations
VAS: Visual analogue scale
